# Podiatrists’ views of assessment and management of pain in diabetes-related foot ulcers: a focus group study

**DOI:** 10.1186/s13047-020-00399-8

**Published:** 2020-06-03

**Authors:** Nicoletta Frescos, Bev Copnell

**Affiliations:** 1 Discipline of Podiatry, School of Allied Health, Human Services and Sport, La Trobe University, Melbourne, 3086 Australia; 2 Northern Clinical School, School of Nursing and Midwifery, Northern Centre for Health Education and Research, College of Science, Health and Engineering, La Trobe University, Melbourne, 3086 Australia

**Keywords:** Diabetes-related foot ulcers, Wound pain, Pain assessment, Pain management

## Abstract

**Background:**

Contrary to the belief that patients with diabetes-related foot ulcers (DRFU) do not experience wound related pain due to the presence of peripheral neuropathy there is increasing evidence that pain can be present. Subsequently, wound-related pain is often underestimated and undertreated. The aim of this study is to describe what influences pain assessment of DRFU.

**Methods:**

A qualitative exploratory study was conducted with podiatrists who managed DRFU. Eight podiatrists were recruited through a professional organisation to participate in a focus group. A thematic analysis was conducted to identify themes that explored the barriers and enablers to pain assessment and management of DRFU.

**Results:**

Three themes emerged. Observational and non-verbal cues were the preferred approaches used to assess wound pain. Assumptions and value judgments of the pain patients experienced and the relationships between podiatrists, patients and other health care practitioners were important influencers on the assessment and management of pain.

**Conclusion:**

The perceived barriers to the assessment and management of wound related pain in DRFU were attitudes and beliefs about pain, lack of DRFU-specific validated assessment tools and lack of knowledge and skills to manage the pain.

## Introduction

Although wounds caused by the pathological processes of diabetes mellitus have in the past been described as usually non-painful it is now becoming more evident that people with diabetic foot wounds can experience wound-related pain [1–4]. When patients with diabetes report wound pain, neuropathic pain is most often the cause; however diabetic foot ulcers can be painful regardless of whether the wound is neuropathic or neuro-ischaemic [3, 5, 6].

Studies have shown that up to 75% of people with diabetic foot ulcers experience wound-related pain [2, 3, 7–10]. Pain occurred while walking or standing, or during the night or when waking up, at dressing changes and also inhibited sleep. No differences were found between neuropathic and neuro-ischaemic wounds in the intensity, type and triggers of wound-related pain.

The complexity of DFRU and the characteristics of wound pain associated with DRFU impacts on accurate pain assessment [2]. Pain can be difficult to assess and describe due to a range of underlying comorbidities such as painful peripheral neuropathy, ischaemia, or other local causes such as inflammation, oedema, foot deformities and abnormal foot biomechanics [3]. The majority of DRFU are neuro-ischaemic in nature [11]. Ischemia can be extremely painful, and neuropathy can mask the pain, and the combination of these pathologies complicates the assessment and therefore an accurate diagnosis of the wound pain may be missed.

When wound pain occurs, it may signal the onset of limb-threatening complications such as critical ischaemia or deep infection. Deep infection can cause wound pain even in the presence of severe neuropathy and thereby prevent or delay wound healing [6, 8, 12]. Another plausible explanation for pain associated with diabetic wounds is excess moisture, which can lead to macerated callus around the edge of the wound, favouring bacterial proliferation which increases pain [13].

Evidence suggest that the reporting of persistent wound related pain is not assessed appropriately by health care practitioners or is dismissed [4, 7, 8, 14–16]. Practitioners often have preconceived presumptions about a patient’s pain based on assumptions related to wound aetiology [5, 17]. There is a misconception that patients with DRFU do not experience wound related pain due to the presence of peripheral neuropathy [5, 6] however, prevalence studies have shown that up to 86% of patients with peripheral neuropathy reported wound-related pain [8, 10, 18].

At present no definitive diagnostic tool or clinical guidelines exist specifically to assess wound-related pain in lower limb wounds which is a significant barrier to the management of pain in patients with chronic wounds [19]. There is an urgent need for the development of evidenced based management strategies to address this serious defect in wound management practice. In order to inform the future development of clinical guidelines to reduce the impact of wound pain on healing and to improve patients’ quality of life and emotional well-being, there is a need to understand the perceptions of health care practitioners on what influences the assessment and management of pain in chronic wounds.

This paper presents the results of a qualitative study of podiatrists working in diabetes foot ulcer management. This was part of a larger mixed methods study with the primary research question of “What is the contemporary status of wound pain assessment and management for clients with chronic lower wounds?”

### Aim

The aim of this focus group study was to explore the barriers and enablers in DRFU pain assessment and the challenges of managing chronic pain in DRFU as perceived by podiatrists.

## Methods

### Ethics

The study was approved by the Faculty of Health Sciences Human Ethics Committee La Trobe University. FHEC No: 11/133 and all participants signed informed consent.

### Study design

A mixed method study was undertaken to obtain an understanding of health care practitioners’ assessment of chronic pain in patients with lower limb wounds and how this understanding is applied to management of pain. A quantitative approach using survey methods was used for the initial phase of the larger study, to explore and describe health care practitioners’ common practice of wound pain assessment. In order to attain the contextual understanding of wound pain assessment and management practices, it was necessary to conduct focus groups with health care practitioners representing various professions working in wound care and in different work settings to further illustrate elements of their wound pain practice. The data obtained from the quantitative phase, as reported in a previous publication [14] were used to inform and guide the approach and the interview questions for the focus groups of health care practitioners and by the clinical and academic experience of the research team.

The focus group enabled participants to explore and clarify their views with active encouragement of group interaction among participants [20]. The facilitator encourages participants to talk to each other, comment on each other’s experiences and points of view [21]. For this study, the focus groups enabled discussion of topics in a “known context” as all participants worked in wound care [22] .

### Sampling and recruitment

A purposive sampling technique was used to recruit participants to enable representation of a diverse and complex range of experiences and particular expertise identified as relevant to the study [20, 23, 24]. This technique adds power to the focus group because it tends to generate rich data and broadly reflects the population from which it is drawn [20]. Four focus groups were conducted representing various health care practitioners working in wound management. Participants for the podiatrists’ focus group were recruited from “high-risk foot” clinics in the greater metropolitan Melbourne area, which specialised in the treatment and management of foot ulcers. Invitation letters were sent via email to the individual potential participants with details of the time and the place of the focus group. These were followed up by phone calls to further discuss the study and confirm attendance. The focus group was conducted at the university campus located in central Melbourne.

### Data collection

The recruited participants received verbal and written information on the study and gave written informed consent prior to the focus group starting.

The focus group interview consisted of a guided in-depth semi-structured interview. The sequence of questions was determined to address the focus group objectives while assisting with the flow of discussion. The interview commenced with broad open-ended questions followed by focal questions. The focus group questions were:

How do you undertake and document wound pain assessment?

What are the barriers to pain assessment?

How can you improve pain assessment?

How do you manage wound pain at (i) wound dressing changes (ii) other times?

What are the barriers to wound pain management?

How can you improve wound pain management?

The focus group lasted approximately 60 min and began by the moderator (NF, a registered podiatrist with  extensive years of experience) asking participants to write their initial responses to the questions on self-adhesive small note paper (Post-it notes). Key words were written on the Post-it notes which were then placed by the participants on the butcher’s paper under each corresponding question. The intention was to ensure that participants had the opportunity to respond to all questions without being influenced by group dynamics or the direction the discussion may take. It also allowed the researcher to monitor the discussion and ensure all points had been covered. This strategy of triangulation of data ensured credibility akin to internal validity of the findings that truly represented the perspectives of the participants [25].

Participants were encouraged to talk and interact with each other and further information was sought where necessary to clarify individual and shared perspectives. Discussions were moderated so that they did not diverge away from the topic of interest and where appropriate discussions on related issues were permitted to explore other aspects to enrich the data. The group’s discussion was audio recorded and transcribed verbatim.

### Data analysis

Thematic analysis was used to analyse the transcribed discussion following the principles of Braun and Clarke [26] which involved a rigorous and systematic approach to the thematic selection.

The first step of this process was familiarisation with the data. NF checked the transcript for errors by listening to the audio files and reading the transcript. The transcript was read at least three times by NF prior to data analysis and observations and comments were written and noted to gain a general sense of concepts or themes that surfaced. This was followed by coding which identified keywords and phrases. An initial coding strategy of line by line coding was undertaken, this was an additional approach to the analysis adopted from Charmaz [27] who states that this approach increases the rigour of analysis. This process was then followed by focus coding to synthesise the most significant and or frequent earlier codes and make the most analytic sense to categorise the data.

The next stage of the analysis was the identification of emergent themes from the coded data, followed by sub-themes, which were categories that grouped codes with similar content. Once the initial coding had been completed, the categories were reviewed and collapsed into clusters in order to reduce duplication of themes and allow appropriate cross-referencing of themes. Following coding of the transcripts, preliminary categories and themes linking the codes were explored. The codes and categories were rechecked to improve consistency of categorisation across the transcripts. The transcripts were then reviewed once more and recoded in order to explore any evidence of new themes based on the revised coding. This iterative process continued until the point was reached when no new information was emerging.

A review of the conceptual interpretation of the data and the categorisation and theme process was undertaken to ensure consistency of meaning and consensus was reached among the researchers (RN, a registered nurse with extensive clinical experience and conducting qualitative research) and NF. The synthesis of the coded data provided the explanation and elaboration in response to the research question. The identification of themes for relevancy and congruency was independently reviewed by BC (a registered nurse with extensive clinical experience and conducting qualitative research). Then NF and BC further explored the data to map connections between themes. The analysis was reviewed over several drafts and revisions to ensure the results accurately reflected the meaning of the data.

## Results

### Participants

A total of eight podiatrists who worked in various clinical work settings for high-risk foot clinics attended the focus group. The age ranged between 24 and 39 years of age and their experience in wound care ranged from 2.5 years to 15 years of practice.

### Key themes

Three main themes were derived from the data. *Looking for cues* described participants’ approaches to assessing wound pain. *Making assumptions* and *Relationships between podiatrists, health practitioners and patients* described important influencers on the assessment and management of pain.

#### Looking for cues

Participants indicated they assessed pain informally rather than formally. Lack of specific tools was cited as the main reason for undertaking informal assessment and the lack of a clinical protocol hindered formal assessment. One participant indicated that using a standard assessment tool was too time consuming:

“Sometimes that can be a time barrier, you've just got so many people to see and asking them the quick basic question sometimes you think ‘oh, that will do for now because I don't have time to do a two-page sheet for pain assessment”*.*

When scales, such as the Visual Analogue Scale, were used, the participants indicated that interpretation of responses could be problematic. They recognised pain as being a subjective response, and that it was difficult to use any given number on the scale as a basis for management. One participant said:

“They say ‘my pain is the worst I've ever felt’ and I still might get a scalpel out . … . The (pain) scales are not necessarily specific to when you treat them”*.*

Moreover, the degree of pain was not necessarily a reliable indication of any underlying pathology, which further complicated the participants’ interpretation. As one participant said:

“With people that already have a degree of neuropathy, if they're complaining of even mild pain, then that might be massive because you know, their pain threshold would be way up here.”

Pain assessment tools did not assist in distinguishing between wound pain and neuropathic foot pain, which participants indicated required a different approach. The participants highlighted that their assessment of pain was mostly undertaken by talking to patients and asking them specific questions. Several suggested that the podiatrist had to initiate the conversation, as patients would not always volunteer the information: as one participant said:

“I think you need to talk to them about pain, not just presume they’re going to tell you”.

Participants highlighted that this verbal assessment was challenging in a number of circumstances. Some patients had difficulty understanding and therefore responding, due to cognitive impairment or speaking little English. Participants reported that some patients were reluctant to admit to feeling pain. One participant spoke of:

“the tough man syndrome where you're asking them and asking them and you're quite sure that they're in pain …. and then they're saying ‘no, it's not painful, you do what you need to do love’ … and they won't tell you when you ask them”.

As noted by many participants, patients might not report pain because of fear of consequences such as amputation*:*

“a lot of it is people don't want to get body parts amputated, so they will put up with a lot.”

Hence, verbal assessment was often limited, and participants would look for other cues such as facial expressions, guarding, or other physical responses: “A kick is usually good”.

#### Making assumptions

Participants indicated that assessment and management of pain were influenced by a range of assumptions, made by podiatrists and other health professionals. Assumptions were made about the presence of pain, whether or not it could be managed and by whom, and the significance of pain assessment and management. Underpinning many of these assumptions were lack of knowledge about pain, resulting in a lack of practitioner competence in managing it.

Most participants equated peripheral neuropathy with inability to feel pain, with comments such as “most of our patients are neuropathic so they don’t feel the pain”, but this view was not held by all. Some participants suggested pain was seen as inevitable and not amenable to intervention:

“They're neuropathic, they're diabetic, there is nothing we can do about it. Control your sugars, take a few Panadol, off you go.”

Participants suggested that many health professionals saw chronic wound pain as unimportant, failing to take it seriously and thus not managing it properly. Several of them spoke of patients’ complaints of pain being “brushed aside”. This resulted in the patient also not taking the pain seriously and ceasing to complain about it, even denying they had pain when asked about it. One participant contested this view, stating:

“Wound pain has a direct impact on wound healing, and we know that increased pain levels or unaddressed pain can have a direct impact on that. So, I can't imagine why you would ignore it.”

Another suggested that the issue was brushed aside because of lack of knowledge, not just of the significance of pain, but of how to manage it:

“It's practitioner knowledge. It comes back to the competency of people knowing about pain. What is pain, and how to treat pain. That's a big one. I don't think people – we don't learn much about pain. We might in Physiology 101 or whatever. I don't remember it, but only from my self-directed learning, that I've gone to look into this further.”

Assumptions about the focus of work were seen to influence pain assessment and management. As one participant said:

“Somebody in the care team has to take ownership of the pain, and I suppose pain assessment has to be prioritised, otherwise it won't happen at all.”

Some participants saw pain as less of a priority in their work and did not take on this ownership of the management of pain:

“We do pass the buck. We assume that the doctor is looking after the pain.”

“It's more about managing the disease than managing the pain through other methods.”

Other participants contested this attitude; as one participant said:

“Of course it's your role, and it's about case management about the patient and the total care of that patient…I can't just ignore that they've got like no blood flow, can I, so why can we ignore they've got pain?”

Participants’ assumptions about patients with diabetes appeared to colour their attitudes to pain assessment and management. Several spoke of encountering patients who were non-compliant with medications or other forms of management.

“…the type of personality that has ignored something or hasn't looked after themselves and is not big into medical intervention or compliance.”

People who were inactive were seen as being less likely to seek, or indeed need, treatment. Specific personality types were met with scepticism:

“They pop in and they've got pain coming from everywhere and you are just not sure whether it's real or not and it's hard to take seriously.”

#### Relationships between podiatrists, health professionals and patients

Patients’ access to effective pain management was influenced by the professional relationships between podiatrists and other healthcare providers, and between patients and health professionals. Participants identified elements of relationships that could either facilitate or hinder effective management.

Participants suggested that pain assessment and management was more likely to be successful if undertaken by a multidisciplinary team. This was particularly relevant if patients required medication, which podiatrists were unable to prescribe. Some participants worked in such a situation, taking part in consultation jointly with medical practitioners. Others spoke of having good relationships with patients’ doctors, which promoted a team approach. Others were less fortunate and spoke of working in isolation and lacked access to medical practitioners.

Appropriate management could also be hindered by hierarchical relationships. Participants trying to refer patients to specialist pain clinics often encountered obstacles, it was highlighted that an appointment was easier to be obtained if the referral was from a medical practitioner.

Patients’ relationships with health professionals – their doctors, other medical staff and the podiatrists themselves – were also seen to influence pain management, and specifically patients’ compliance. Here the issue was mainly around trust; if patients did not trust the staff they were less likely to discuss their pain with them in detail. Conversely, management was improved if the patient and health care provider worked together as a team, which required commitment from both parties. As one participant explained:

“I think it's identifying with the patient that pain is a patient goal, like, to actually be pain free would be a nice thing for them …. So if the patient can identify that is a goal of theirs …. you've got to get the patient buy-in. …. So if you make it more goal centred and patient centred care it might work.”

## Discussion

The aim of this study was to identify from podiatrists’ perspective the barriers and enablers in DRFU pain assessment and management. The study identified several barriers as impacting on accurate assessment and management of pain in DRFU which were attributed to podiatrists’ work practices and knowledge, health care professional relationships and patient issues.

The study identified that the preferred method for wound pain assessment was by the patients’ body language or non-verbal cues and talking to patients and asking specific questions. This supports the findings of a survey of wound care practitioners in Australia, which included podiatrists, that the most common approach in identifying and assessing pain was talking to the patient and asking the patient to give a self-report rating of their pain [14]. Generally, there is a tendency by health care practitioners to not use a valid pain assessment tool but place greater reliance on the observation of the patient’s body language, appearance, what they express and non-verbal cues [28, 29].

Validated pain assessment tools were not used as there was no specific tool to assess pain in DRFU nor clinical guidelines or protocols deemed suitable. Although there are multiple pain assessment tools available, only four common pain measurement tools have been identified to be suitable for wound related pain, however there is insufficient evidence to recommend one pain assessment tool that is suitable for lower limb wounds.

Using pain scales was described as problematic as they did not distinguish between wound pain and neuropathic or ischaemic foot pain. Furthermore, obtaining appropriate ratings was perceived as difficult, as patients had difficulties in converting experienced pain into numbers, a common finding in many pain assessment studies [28, 30]. Assessing pain in patients with cognitive impairment and patients who could not speak English was a barrier considered as a major challenge by all the practitioners. To overcome this, observed non-verbal cues such as the patient’s body language, guarding of the wound and facial expressions were used to assess wound pain. Podiatrists also described the challenges of interpreting the patient’s ratings when they did not correspond with the clinical observation. These findings are comparable to a study by Young et al [31] on nurses’ perceptions and attitudes towards pain assessment.

More importantly, a key factor impacting on the assessment and management of wound-related pain was the assumption that people with neuropathy do not feel pain, or their symptoms are related to neuropathy or vascular disease; hence, the podiatrists do not assess wound pain. This reflects the findings of Sibbald et al [6] of the misconception that patients with DRFU do not experience wound pain. This assumption is an important finding as there is increasing evidence in the literature confirming that patients with diabetic foot ulcers experience pain sensations regardless of whether the wound is neuropathic or neuro-ischaemic [2, 3, 10].

Although the assessment of pain was one element of the problem, a compounding problem was frustration of not knowing what to do with the assessment results. Many did not regularly assess pain as they could not treat it. Furthermore, the importance of the identification and management of wound pain is not well understood by podiatrists and this was deemed to be one of the main issues in pain management. Although health care practitioners have knowledge of wound care, many studies of wound pain have concluded that health care practitioners involved in wound management lack some knowledge and understanding of pain in wound healing [3, 32, 33]. A report on chronic wounds in Australia stated that the lack of confidence, skills and knowledge is due to a lack of education in undergraduate, post graduate and professional development and there is a need for more education and training in evidence based wound care [34]. The results of the present study support these conclusions that the lack of knowledge is a major barrier to effective pain management.

The reluctance by patients to report pain was suggested to be attributed to the common assumption that wound pain is normal and must be tolerated. Most important was the fear of reporting pain, which was driven by the beliefs and perceptions of subsequent sequelae of events such as the fear having their leg amputated. A study by Bengtsson et al [3] suggested that patients with sensory neuropathy are often reluctant to talk about their pain for fear they will be referred for amputation. Studies on pain in the older person have shown fear is a contributing factor to the denial of pain [35].

Relationships between patients and health care practitioners were reported to influence the accuracy of wound pain assessment and management. Trust and rapport were identified by the podiatrists as a determinant of patients’ compliance with the management of their wound. The patients’ trust was related to the confidence they had in their practitioner’s care and management strategies. It was important to develop mutual trusting relationships with patients and work together in decision making that can lead to improved outcomes [36]. Although this has been reported extensively in the literature for doctor or nurse patient relationship, no studies have been conducted on podiatrist-patient relationship and trust.

A multidisciplinary team approach and collaboration between podiatrists and medical practitioners was professed as imperative to improve patients’ experiences in receiving wound care to achieve positive outcomes. The benefits of team approach to DRFU has been well documented in the literature as best practice for the prevention of lower extremity amputations and enhancement of health related quality of life [37].

Value judgements and personal bias based on podiatrists’ perception of patients’ behaviour or self-care influenced the assessment of patients’ wound pain. Patients who were difficult or non-compliant were stereotyped or labelled as a “*type of personality*”. While not previously described among podiatrists, this is a common finding in studies on doctor-patient relationships and social evaluation of their patients [38]. A study by May et al [39] found that doctors quickly make evaluative judgements on patients’ motives, legitimacy of their symptoms and congruence between the doctor’s and the patient’s conceptual model of illness, which reflects similar behaviour of podiatrists to their difficult patients. Furthermore, Hill [38] states that stereotypical thinking and moral judgements increase when workload is high and there are limited resources, which may also be a plausible reason for the podiatrists’ attitude.

In summary, the barriers reported by the podiatrists such as workload requirements, time pressure, attitudes and beliefs about pain, and lack of knowledge and skills to manage the pain are similar to other published studies on pain management in general [35, 40]. Furthermore, these perceived barriers are consistent with the theory proposed by Smith et al [41], of habituated behaviours of health professionals, which suggests that health professionals’ behaviour is shaped by beliefs or contextual factors such the characteristics of a condition or illness, external policy and organisational support, and lack of knowledge.

### Limitations

There are several disadvantages of a focus group that should be taken into consideration. Firstly, participants may have felt inhibited in expressing their honest views or felt discouraged from contributing to the conversation to express their opinions in a group environment. Secondly, the level of experience of the participants was varied; some participants may have felt they did not have extensive experience or may have been inhibited by the hierarchical positions of other participants. Homogeneity in a focus group is important for participants to feel equal; however, it is also important to avoid excessive homogeneity in order to encourage the collection of different points of view [42].

## Conclusion

The management of pain in patients with DRFU is a significant problem and should not be ignored. This study has provided an increased understanding of the perceived barriers in the assessment and management of wound-related pain in patients with DRFU. The findings identify the importance of providing education and improvement in knowledge of wound-related pain for both patients, podiatrists and health care practitioners to change attitude and behaviour, to acknowledge that the presence of pain in DRFU is important and must be managed. Furthermore, there is a need for the development of a specific pain assessment tool and clinical guidelines for DRFU to manage wound-related pain effectively, in order to reduce the impact of wound pain on healing, prevent amputation and to improve patients’ quality of life.

## Data Availability

The dataset used and analysed during the current study are available from the corresponding author on reasonable request.

## Abbreviations

DRFU: Diabetes-related foot ulcers
